# Clinical and electrocardiogram presentations of patients with high serum potassium concentrations within emergency settings: a prospective study

**DOI:** 10.1186/s12245-022-00422-8

**Published:** 2022-05-26

**Authors:** Liqaa A. Raffee, Khaled Z. Alawneh, Muhannad J. Ababneh, Heba H. Hijazi, Rabah M. Al abdi, Mahmoud M. Aboozour, Fadi A. Alghzawi, Abdel-Hameed Al-Mistarehi

**Affiliations:** 1 Department of Accident and Emergency Medicine, Faculty of Medicine, Jordan University of Science and Technology, P.O. Box 630001, Irbid, 22110 Jordan; 2 Department of Diagnostic Radiology and Nuclear Medicine, Faculty of Medicine, Jordan University of Science and Technology, Irbid, Jordan; 3 Division of Cardiology, Department of Internal Medicine, Faculty of Medicine, Jordan University of Science and Technology, Irbid, Jordan; 4 Chair of Department of Health Services Administration, College of Health Sciences, University of Sharjah, Sharjah, United Arab Emirates; 5 Department of Health Management and Policy, Faculty of Medicine, Jordan University of Science and Technology, Irbid, Jordan; 6 Department of Biomedical Engineering, Faculty of Engineering, Jordan University of Science and Technology, Irbid, Jordan; 7 Department of Public Health and Family Medicine, Faculty of Medicine, Jordan University of Science and Technology, P.O. Box 630001, Irbid, 22110 Jordan

**Keywords:** Serum potassium, Hyperkalemia, Electrocardiogram, ECG, Sensitivity

## Abstract

**Background:**

Elevated potassium level is a common and reversible peri-arrest condition. Diagnosis and management of hyperkalemia in a short time is critical, where electrocardiogram (ECG) alterations might be helpful. We aimed to investigate the role of clinical features and ECGs in early diagnosing and treating hyperkalemia.

**Methods:**

Prospectively, adult patients who presented to the emergency department (ED) from July 2019 to March 2020 with hyperkalemia (serum potassium ≥5.5mmol/L) were included. History was obtained, and laboratory investigations and ECGs were performed at the presentation and before initiating hyperkalemia therapy. Hyperkalemia severity was divided into mild (5.5–5.9mmol/L), moderate (6.0–6.4mmol/L), and severe (≥6.5mmol/L). A cardiologist and emergency physician blinded to laboratory values, study design, and patients’ diagnoses interpreted ECGs and presenting symptoms independently to predict hyperkalemia.

**Results:**

Sixty-seven hyperkalemic patients with a mean (±SD) serum potassium level of 6.5±0.7mmol/L were included in this study. The mean age was 63.9±15.1, and 58.2% were females. Hyperkalemia was mild in 10.4%, moderate in 40.3%, and severe in 49.3%. Almost two thirds of patients (71.6%) had hypertension, 67.2% diabetes, and 64.2% chronic kidney disease. About one-quarter of patients (22.4%) were asymptomatic, while fatigue (46.3%), dyspnea (28.4%), and nausea/vomiting (20.9%) were the most common presenting symptoms. Normal ECGs were observed in 25.4% of patients, while alterations in 74.6%. Atrial fibrillation (13.4%), peaked T wave (11.9%), widened QRS (11.9%), prolonged PR interval (10.5%), and flattening P wave (10.5%) were the most common. Peaked T wave was significantly more common in severe hyperkalemia (87.5%) than in mild and moderate hyperkalemia (12.5%, 0.0%, respectively) (*p=0.041*). The physicians’ sensitivities for predicting hyperkalemia were 35.8% and 28.4%, improved to 51.5% and 42.4%, respectively, when limiting the analyses to severe hyperkalemia. The mean (±SD) time to initial hyperkalemia treatment was 63.8±31.5 min. Potassium levels were positively correlated with PR interval (*r*=0.283, *p=0.038*), QRS duration (*r*=0.361, *p=0.003*), peaked T wave (*r=0.242*, *p=0.041*), and serum levels of creatinine (*r*=0.347, *p=0.004*), BUN (*r*=0.312, *p=0.008*), and CK (*r*=0.373, *p=0.039*).

**Conclusions:**

The physicians’ abilities to predict hyperkalemia based on ECG and symptoms were poor. ECG could not be solely relied on, and serum potassium tests should be conducted for accurate diagnosis.

## Background

Elevated serum potassium level above the upper limit of normal is a common electrolyte imbalance in adults that might be a life-threatening condition causing lethal cardiac arrhythmias; thereby, this condition requires immediate management [1–3]. The incidence rates of hyperkalemia vary among studies, with estimates ranging between 1.1 and 10% among hospitalized patients [4–7]. Emergency department (ED)-based studies reported incidence rates between 0.36 and 3.6% of hyperkalemia [8–11]. The development of new oral therapies for hyperkalemia has led to renewed interest in hyperkalemia [12, 13]. Also, the increased use of hyperkalemia-inducing drugs, such as antihypertensive drugs including angiotensin-converting enzyme inhibitors (ACEIs), angiotensin-receptor blockers (ARBs), and beta-blockers, as well as spironolactone has led to increasing the incidence rates of hyperkalemia among ED patients [14–16]. Besides, as more patients live with chronic kidney disease, renal failure, hemodialysis, and congestive heart failure (CHF), hyperkalemia cases will trend to increase [11, 17, 18].

The recognition of patients with hyperkalemia in ED is challenging. Even though severe hyperkalemia might be associated with cardiac arrest or muscle paralysis, the symptoms in most hyperkalemia cases are non-specific [19–21]. These symptoms may include chest pain, palpitations, weakness, muscle spasm, numbness, tingling, trouble breathing, dysphagia, abdominal pain, fatigue, nausea, or vomiting. Also, the physical findings of absent or depressed deep reflexes, hypotension, or dysrhythmia are not specific for hyperkalemia diagnosis [19–21]. Thus, history and physical examination in patients with high potassium levels could not be enough to diagnose hyperkalemia, and the initiation of appropriate treatment for hyperkalemia might be delayed due to the non-specificity of presentation [22].

The electrocardiogram (ECG) is an inexpensive, non-invasive, highly compliant, broadly available, and readily accessible test. Theoretically, abnormally elevated serum potassium levels above 6.0 mmol/L may be associated with apparent changes in ECG, including peaked T waves, prolonged intraventricular and atrioventricular conductions, the disappearance of the P waves, QRS prolongation, depression, and obliteration of the ST segments, and shortening of QT corrected (QTc) interval [22–28]. Thus, ECG was suggested by some clinicians and researchers as a good indicator of hyperkalemia, especially in critical situations and among unstable patients [20, 29–32]. Also, it has been reported that the ECG changes became more evident with severely increased serum potassium levels, and a better prediction of hyperkalemia using ECG was noted as the hyperkalemia severity increases [31, 33, 34]. The reliability of ECG changes in diagnosing hyperkalemia is clinically unclear [24, 35, 36]. There is no clear evidence to demonstrate high sensitivity or specificity of the ECG changes in predicting severe hyperkalemia incidents with more than 6.0 mmol/L potassium levels [33]. Moreover, ECG changes could hardly be observed when the concentrations of serum potassium levels are slightly elevated above the normal level [33, 34, 37].

A previous experiment conducted by Porter et al. and aimed to predict hyperkalemia in dogs using ECG parameters incorporated with an artificial neural network [38]. This neural network had a high sensitivity (89%), specificity (77%), and accuracy (86%) with a positive likelihood ratio of 3.9 [38]. However, these results have not been reproduced in human clinical settings. Multiple investigators extensively studied the ECG alterations suggestive of hyperkalemia clinically and indicated that ECG findings were variable, unreliable, and had poor sensitivity clinically [22, 32, 33, 39]. Wrenn et al., in their retrospective study, asked two independent emergency physicians to predict the presence or absence of hyperkalemia and its severity based on the interpretation of ECGs solely [32]. They were blinded to the laboratory serum potassium values, patients’ clinical diagnoses, and each other’s reading. Their sensitivities in the ECG diagnosis of hyperkalemia were low to be estimated as 34 to 43%. While the specificities were higher and ranged between 85 and 86%, with a high degree (90%) of agreement between the readers [32].

Interestingly, Wrenn et al. observed improvements in the readers’ sensitivities to become between 55 and 62% when the readers were limited to interpret ECGs with serum potassium levels of more than 6.5 mmol/L [32]. Thus, most missed hyperkalemia diagnoses occurred with serum potassium levels lower than 6.5 mmol/L. Also, the authors suggested that hyperkalemia management in the ED could be initiated solely based on ECG abnormalities in unstable patients, while it could be delayed among stable patients until laboratory confirmation of high potassium levels [32].

Although the recommendations of immediate therapy for any patient with high serum potassium level [40] and the initial management of hyperkalemia involves few maneuvers, previous studies reported an average delay of 2 h before initiation of treatment of severe hyperkalemia [2, 22]. Given these uncertainties and the necessity for rapid management of hyperkalemia to decrease its life-threatening associated risks, it is crucial to understand the fastest diagnostic tool for hyperkalemia. We hypothesized that clinical and ECG findings would be associated with early diagnosis and initiation of hyperkalemia management. Thus, the primary goal of this study was to describe the presenting symptoms, clinical features, and initial ECG parameters associated with laboratory diagnosis of hyperkalemia among ED patients. Also, we investigated the ability of two experienced independent physicians to predict hyperkalemia based on initial ECG alterations and presenting symptoms. Thus, investigating the ECG alterations suggestive of hyperkalemia and clinical presentation as a possible screening tool for the purpose of early diagnosis and management of hyperkalemia. Besides, we aimed to identify the determinants of time from triage to the initiation of hyperkalemia management in ED.

## Materials and methods

### Study design, settings, and participants

Prospectively, all adult patients presented to the ED of King Abdullah University Hospital (KAUH), a tertiary hospital in North of Jordan affiliated with Jordan University of Science and Technology (JUST), during the nine months of July 2019 to March 2020 with laboratory diagnosis of hyperkalemia were included in this study. There is no universal definition for hyperkalemia. Thus, hyperkalemia was identified as a serum potassium level higher than or equal to 5.5 mmol/L as the hospital laboratory defined normal potassium levels between 3.5 and 5.4 mmol/L. Cases included in this study were required to have a documented non-hemolyzed plasma or serum potassium level ≥5.5 mmol/L with a coincident ECG recorded within 1 h of laboratory draw and before hyperkalemia therapy. Written informed consents for participation were obtained from all participants. Predetermined study exclusion criteria were the age of younger than 18 years, lack of documented hyperkalemia, hemolyzed blood sample, absence of a coincident ECG within 1 h of hyperkalemia episode, and any patient with a baseline-paced ECG limiting assessment of ECG changes. Patients with normal repeated potassium values in case of repeated test conduction and those who did not receive hyperkalemia therapy were also excluded. In addition, patients who refused to participate and those with cardiac arrest before ED arrival were excluded as cardiopulmonary resuscitation was reported to be a secondary cause of hyperkalemia [41, 42].

### Data collection and processing

Detailed history taking and physical examination were performed at the time of patient presentation to the ED. Demographic characteristics, including age, gender, and smoking status, chief complaint, presenting symptoms, comorbid diagnoses, presence of underlying kidney disease, regular dialysis, chronic medications’ use, the place where the patient came from (home/another clinic), and the mode of transmission to the ED (walking/private car/ambulance) were obtained from the patients and documented. Also, electronic medical records, medical charts, and pharmacy records for all identified cases were reviewed in detail. Vital signs including temperature, heart rate, respiratory rate, blood pressure, and O_2_ saturation using a pulse oximeter were recorded for participants at the time of presentation. The time to hyperkalemia treatment was calculated by the difference between the triage time and the first hyperkalemia therapy administration by the ED nurse staff.

Blood samples were obtained by the nursing staff of ED and sent to the central hospital laboratory for analysis. The hospital laboratory staff posts the results of the specimens on the computer database system of the hospital to be immediately accessible by medical staff. The study conducted several laboratory tests, including serum potassium, creatinine, blood urea nitrogen (BUN), creatine kinase-MB (CK-MB), and creatine kinase (CK) levels, arterial blood gas results, and troponin as a biomarker of cardiac injury. The laboratory data were obtained from the electronic medical record.

Potassium values were determined as part of the chemistry panel. As part of the hospital protocol and in the case of abnormal potassium values, the laboratory team directly informs the responsible emergency physician by a telephone call within 5 min of getting the potassium laboratory result. Initial laboratory-confirmed potassium values were recorded and included in the database of this study. Based on potassium levels, hyperkalemia severity was divided into mild (5.5–5.9 mmol/L), moderate (6.0–6.4 mmol/L), and severe (≥6.5 mmol/L) based on European Resuscitation Council Guidelines and previous studies [43–45].

Along with laboratory samples, ECGs were performed simultaneously by trained research assistant nurses. No hyperkalemia therapy was administered before the conduction of the ECG and blood sample drawing. After that, all the initial ECGs were read and interpreted independently by two experienced faculty physicians in real-time; one is a board-certified internist and cardiologist, while the other has a board certification in emergency medicine. Although both readers were aware of the study objectives and the patients’ presenting symptoms, they were blinded to all laboratory values, the study design, patients’ diagnoses and comorbidities, and each other’s readings. Also, neither reader was a caregiver for any of the patients. Old ECGs and ECG tracings within the emergency admission were not recorded and not available to interpret the initial ECGs. The readers documented the observed ECG changes suggestive of hyperkalemia and their opinions about the presence or absence of hyperkalemia based on the initial ECGs and patients’ symptoms at the time of ED presentation.

The ECG criteria used to predict hyperkalemia as documented by the readers included flattening or absence of the P wave, prolonged PR interval (>200ms), widened QRS complex (>120ms), and “peaked T wave” which is an increased T wave amplitude with a narrow base and defined as a pointed, symmetrical, narrow, and peaked T wave with amplitude taller than one large square in the limb leads or two large squares in the chest leads. Also, ST-segment elevation or depression, right and left bundle branch blocks (RBBB and LBBB), arrhythmias, and shortened QTc interval (<350ms for males and <360ms for females) were investigated and documented by the readers. The duration values of PR intervals, QRS complexes, QTc intervals, and RR intervals were calculated and documented in milliseconds. The QTc interval was calculated by using Bazett’s formula:

$$\mathrm{QTc}=\mathrm{QT}\ \mathrm{intervals}\ \mathrm{in}\ \mathrm{seconds}/\surd \mathrm{cardiac}\ \mathrm{cycle}\ \mathrm{in}\ \mathrm{seconds}=\mathrm{QT}/\surd \mathrm{RR}$$

### Ethical considerations

All procedures performed in this study involving human participants were reviewed and ethically approved by the Institutional Review Board (IRB) of the research and ethics committee at JUST and KAUH, where the study took place (IRB approval number of 26/122/2019). This study was conducted following the 1975 Helsinki Declaration, as revised in 2008 and its later amendments or comparable ethical standards. To keep the confidentiality of participants, all information was deidentified; a patient-specific unique number was created for each patient, and this identification file that links the patient name and hospital number to the newly created number had been locked and password protected. All analysis and further work were performed on a deidentified file. Part of this study data and results were presented as an abstract and poster in the 70th Annual Scientific Session and Expo of the American College of Cardiology (ACC) in 2021 [46].

### Statistical analysis

The data was analyzed using the IBM Statistical Package for the Social Sciences (SPSS) windows software, version 25.0. Categorical variables were displayed in frequency and percentage, while continuous variables were reported using mean ± standard deviation (±SD). A chi-square test or Fisher’s exact test was used to assess the differences of mildly, moderately, and severely hyperkalemic patients in regards to ECG criteria, presenting symptoms, and medications’ use. The ECG readers’ sensitivities in detecting hyperkalemia were measured, and kappa (κ) was calculated as an inter-rater agreement measure between the readers. Bivariate correlation using the Pearson correlation coefficient (r) test was conducted to assess the correlations of the time to hyperkalemia treatment and serum potassium levels with the ECGs criteria, including PR intervals, QRS complexes, QTc intervals, RR intervals, and the presence of peaked T wave (scored with one when it is present and zero when it is absent). Also, the correlations of the time to hyperkalemia treatment and serum potassium levels with the patients’ vital signs and laboratory data were measured using the Pearson correlation coefficient (r) test.

## Results

A total of 67 hyperkalemic patients were included in this study. The participants’ mean age was 63.9 ± 15.1, and more than half (58.2%) were females. The mean (±SD) serum potassium level of patients was 6.5 ± 0.6 mmol/L and ranged between 5.6 and 9.0 mmol/L. Hyperkalemia was mild in seven (10.4%) patients and moderated in 27 (40.3%), while approximately half of the patients (*n*=33, 49.3%) had severe hyperkalemia. Table 1 summarizes the patients’ demographic and clinical characteristics. Most hyperkalemic cases (*n*= 61, 91%) had at least two comorbid chronic diseases. Almost two thirds of the hyperkalemic patients (71.6%) had hypertension (HTN), 67.2% had diabetes mellitus (DM), and 64.2% suffered from chronic kidney disease (CKD). About one third of participants (23.9%) were on regular dialysis. Regarding medications, high percentages of hyperkalemic patients took statins (64.2%), proton pump inhibitors (PPIs) (58.2%), non-steroid analgesics (53.8%), beta-blockers (52.2%), calcium channel blockers (CCBs) (41.8%), ACEIs (31.3%), and ARBs (28.4%).

**Table 1:** The baseline demographic and clinical characteristics of patients with hyperkalemia

Variables		Frequency (%)
**Gender**	Male	28 (41.8)
	Female	39 (58.2)
**Age (mean ± SD):** 63.90 ± 15.13		
**Smoking status**	Current smoker	15 (22.4)
	Ex-smoker	8 (11.9)
	Non-smoker	44 (65.7)
**Place the patient came from**	Home	49 (73.1)
	Transfer from another clinic	18 (26.9)
**Mode of transport to the emergency department**	Walking	18 (26.9)
	Private car	30 (44.8)
	ambulance	19 (28.4)
**Comorbidities**	HTN	48 (71.6)
	DM	45 (67.2)
	Chronic renal disease	43 (64.2)
	IHD	17 (25.4)
	CHF	13 (19.4)
	Hypothyroidism	6 (9.0)
	Stroke	5 (7.5)
	Gout	5 (7.5)
	Arterial fibrillation	5 (7.5)
	Venous thromboembolism	4 (6.0)
	COPD	1 (1.5)
**Patients on regular dialysis**		16 (23.9)
**Medications**	ACEIs	21 (31.3)
	ARBs	19 (28.4)
	Beta-blockers	35 (52.2)
	CCBs	28 (41.8)
	Aspirin	34 (50.8)
	Other NSAIDs	2 (3.0)
	LMWH or unfractured heparin	5 (7.5)
	Warfarin	5 (7.5)
	PPIs	39 (58.2)
	Statins	43 (64.2)
	Metformin	12 (17.9)
	Insulin	23 (34.3)
	Levothyroxine	6 (9.0)
	Allopurinol	4 (6.0)
	Cortisol	5 (7.5)
	Loop diuretics	15 (22.4)
	Thiazide diuretics	7 (10.5)
	Potassium-sparing diuretics	3 (4.5)
	Digitalis	2 (3.0)
	Clopidogrel	8 (11.9)

*Abbreviations: HTN* Hypertension, *DM* Diabetes mellitus, *IHD* Ischemic heart disease, *CHF* Congestive heart failure, *COPD* Chronic obstructive pulmonary disease, *ACEIs* Angiotensin-converting enzyme inhibitors, *ARBs* Angiotensin-receptor blockers, *CCBs* Calcium channel blockers, *NSAIDs* Non-steroidal anti-inflammatory drugs, *LMWH* Low molecular weight heparin, *PPIs* Proton pump inhibitors

**Fig. 1:**
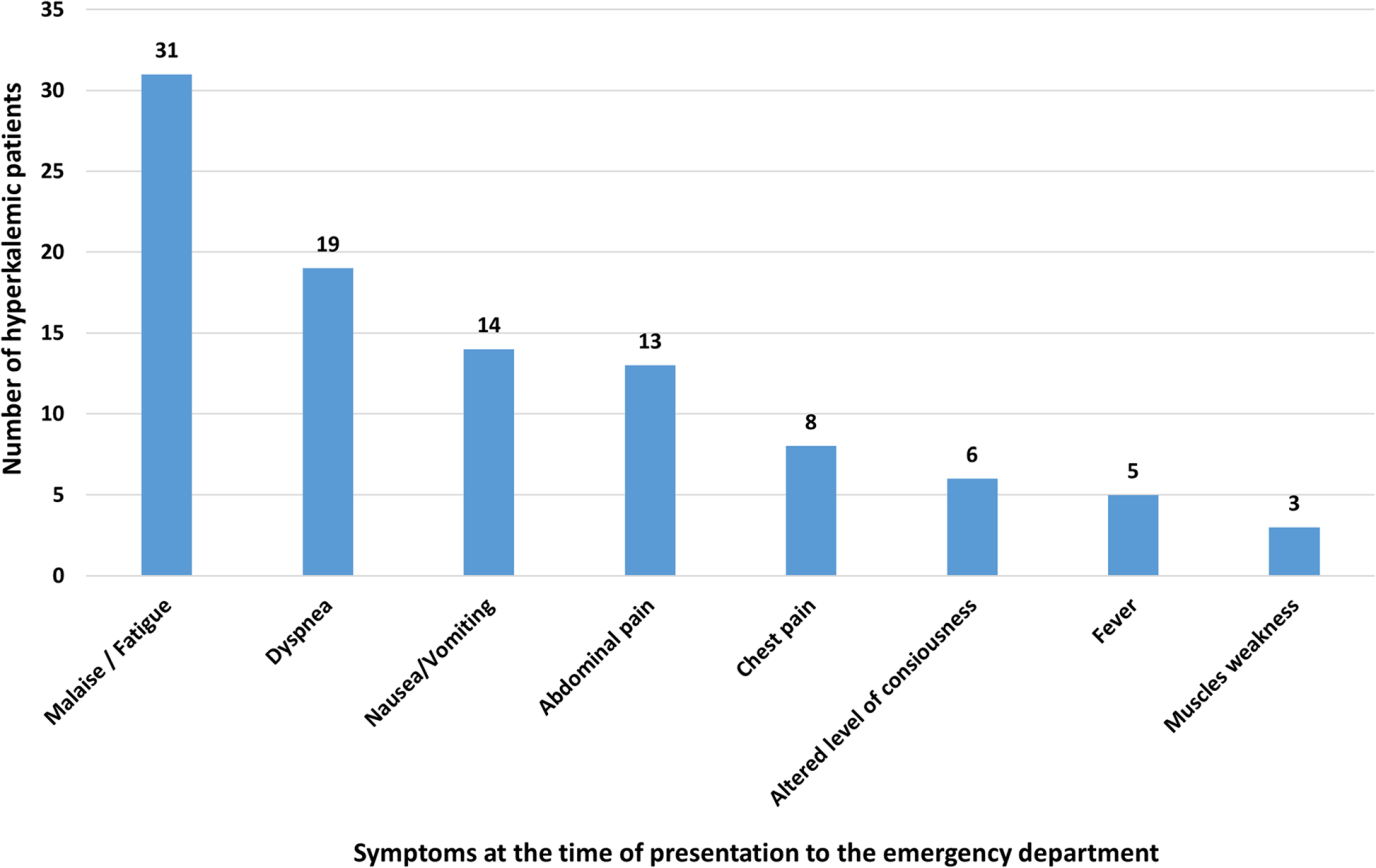
Presenting symptoms associated with laboratory diagnosis of hyperkalemia among emergency department (ED) patients

About one-quarter of hyperkalemic patients (*n*=15, 22.4%) were asymptomatic, and hyperkalemia was an incidental finding. The most common presenting symptoms in the symptomatic patients were fatigue/malaise (46.3%), dyspnea (28.4%), nausea/vomiting (20.9%), abdominal pain (19.4%), and chest pain (11.9%). While the least presenting symptoms associated with hyperkalemia were altered level of consciousness (9.0%), fever (7.5%), and muscle weakness (4.6%). There were no significant differences in presenting symptoms between mild, moderate, and severe categories of hyperkalemia (*p>0.05*). Presenting symptoms of hyperkalemic patients can be seen in Fig. 1. Oxygen saturation below 94% was observed among 31.3% of hyperkalemic patients at the time of presentation. Most of the hyperkalemic patients had high levels of creatinine (94%) and BUN (91%), and approximately two thirds of hyperkalemic patients (70.9%) had metabolic acidosis. Vital signs and laboratory data are shown in Table 2.

**Table 2:** Vital signs and laboratory values of hyperkalemic patients at the time of emergency presentation

Vital signs at the time of presentation	Frequency (%)
Fever (≥38°C)	5 (7.5)
Tachycardia (>100 beats per min)	3 (4.5)
Tachypnea (> 20 breaths per min)	8 (11.9)
Hypotension (< 90/60)	6 (9.0)
Elevated blood pressure (> 139/89)	14 (20.9)
Low O_2_ saturation (<94%)	21 (31.3)
**Laboratory results**^**a**^	**Frequency (%)**
High creatinine (> 115 μmol/L for males and >97 μmol/L for females)	63 (94.0)
High BUN (>7.14 mmol/L)	61 (91.0)
High troponin (> 0.02 ng/ml)	22 (32.8)
High CK-MB (> 24 IU/L)	14 (20.9)
High CK (> 190 units/L)	11 (16.4)
**Metabolic disorders (based on ABG)**^**b**^	**Frequency (% out of 55)**
Metabolic acidosis	39 (70.9)
Respiratory acidosis	2 (3.6)
Mixed acidosis	3 (5.5)
No acid abnormality	11 (20)

*Abbreviations*: *BUN* Blood urea nitrogen, *CK-MB* Creatine kinase-MB, *CK* Creatine kinase, *ABG* Arterial blood gas

^a^Abnormal cutoff points were predetermined based on the involved hospital laboratory reference of normal ranges

^b^Arterial blood gas test was not performed in 12 patients

Normal ECGs were observed in 17 (25.4%) hyperkalemic patients, while abnormalities were recorded in 50 (74.6%) patients. Of those, 8 ECGs (11.9%) had peaked T waves, 8 ECGs (11.9%) had widened QRS complexes, 7 ECGs (10.5%) had prolonged PR intervals, 7 ECGs (10.5%) had flattening P waves, 4 ECGs (6.0%) had ST-segment depressions, 3 ECGs (4.5%) had RBBB, 2 ECGs (3.0%) had disappearance of P waves, and 2 ECGs (3.0%) had LBBB. In contrast, shortened QTc intervals and ST-segment elevations were observed in 1 ECG (1.5%) for each. Atrial fibrillation was the most common arrhythmia observed among hyperkalemic patients (*n*=9, 13.4%), followed by sinus tachycardia (*n*=8, 11.9%), sinus bradycardia (*n*=6, 9.0%), and the least common one was Supraventricular tachycardia (SVT) (*n*=1, 1.5%).

The frequencies of ECG alterations by hyperkalemia severity are shown in Table 3. Peaked T waves abnormality was significantly more common in severe hyperkalemia (87.5%) than in mild and moderate hyperkalemia (12.5%, 0.0%, respectively) with a *p-value of 0.041*. In comparison, none of the other ECG changes suggestive of hyperkalemia was significantly differed by the severity of hyperkalemia (*p>0.05*). Table 4 shows the chronic medications used by the patients and the hyperkalemia severity. The severity of hyperkalemia was not significantly differed by the types of medications.

**Table 3:** The frequency of ECG changes suggestive of hyperkalemia in mildly, moderately, and severely hyperkalemic patients

ECG changes suggestive of hyperkalemia	Hyperkalemia severity			Chi-square, ***p-value*** (***X***^**2**^, degree of freedom)
	Mild (5.5–5.9 mmol/L) (*N*=7) *N* (%)	Moderate (66.4 mmol/L) (*N*=27) *N* (%)	Severe (≥6.5 mmol/L) (*N*=33) *N* (%)	
Flattening of P wave	1 (14.3)	2 (7.4)	4 (12.1)	*0.788* (0.476, 2)
Disappearance of P wave	0 (0.0)	1 (3.7)	1 (3.0)	*0.876* (0.264, 2)
Prolonged PR interval (> 200ms)	0 (0.0)	3 (11.1)	4 (12.1)	*0.733* (2.016, 2)
Widened QRS (>120ms)	1 (14.3)	2 (7.4)	5 (15.2)	*0.642* (0.888, 2)
Peaked T wave	1 (14.3)	0 (0.0)	7 (21.2)	*0.041* (6.396, 2)
Shortened QT-corrected (QTc) interval	0 (0.0)	1 (3.7)	0 (0.0)	*0.471* (1.504, 2)
ST elevation	0 (0.0)	1 (3.7)	0 (0.0)	*0.471* (1.504, 2)
ST depression	1 (14.3)	1 (3.7)	2 (6.1)	*0.756* (0.559, 2)
Right bundle branch block (RBBB)	1 (14.3)	0 (0.0)	2 (6.1)	*0.219* (3.033, 2)
Left bundle branch block (LBBB)	0 (0.0)	0 (0.0)	2 (6.1)	*0.346* (2.124, 2)
Arrythmias				*0.449* (7.846, 8)
Atrial fibrillation	2 (28.6)	2 (7.4)	5 (15.2)	
Sinus bradycardia	0 (0.0)	5 (18.5)	1 (3.0)	
Sinus tachycardia	1 (14.3)	3 (11.1)	4 (12.1)	
Supraventricular tachycardia (SVT)	0 (0.0)	0 (0.0)	1 (3.0)

**Table 4:** The frequency of chronically used medications by hyperkalemia severity

Medications	Hyperkalemia severity			Chi-square, ***p-value*** (***X***^**2**^, degree of freedom)
	Mild (5.5–5.9 mmol/L) (*N*=7) *N* (%)	Moderate (6-6.4 mmol/L) (*N*=27) *N* (%)	Severe (≥6.5 mmol/L) (*N*=33) *N* (%)	
ACEIs	2 (28.6)	7 (25.9)	12 (36.4)	*0.677* (0.780, 2)
ARBs	4 (57.1)	6 (22.2)	9 (27.3)	*0.185* (3.374, 2)
Beta-blockers	3 (42.9)	14 (51.9)	18 (54.5)	*0.853* (0.319, 2)
CCBs	5 (71.4)	11 (40.7)	12 (36.4)	*0.230* (2.939, 2)
Aspirin	4 (57.1)	15 (56.6)	15 (45.5)	*0.693* (0.734, 2)
Other NSAIDs	0 (0.0)	2 (7.4)	0 (0.0)	*0.217* (3.054, 2)
LMWH or unfractured heparin	2 (28.6)	1 (3.7)	2 (6.1)	*0.076* (5.163, 2)
Warfarin	0 (0.0)	2 (7.4)	3 (9.1)	*0.708* (0.691, 2)
PPIs	4 (57.1)	17 (63.0)	18 (54.5)	*0.804* (0.436, 2)
Statins	4 (57.1)	18 (66.7)	21 (63.6)	*0.892* (0.228, 2)
Metformin	0 (0.0)	5 (18.5)	7 (21.2)	*0.411* (1.779, 2)
Insulin	5 (71.4)	10 (37.0)	8 (24.2)	*0.054* (5.851, 2)
Levothyroxine	1 (14.3)	2 (7.4)	3 (9.1)	*0.850* (0.324, 2)
Allopurinol	1 (14.3)	1 (3.7)	2 (6.1)	*0.574* (1.110, 2)
Cortisol	1 (14.3)	2 (7.4)	2 (6.1)	*0.754* (0.566, 2)
Loop diuretics	3 (42.9)	11 (40.7)	1 (33.3)	*0.798* (0.451, 2)
Thiazide diuretics	1 (14.3)	3 (11.1)	3 (9.1)	*0.910* (0.188, 2)
Potassium-sparing diuretics	0 (0.0)	1 (3.7)	2 (6.1)	*0.756* (0.559, 2)
Digitalis	1 (14.3)	0 (0.0)	1 (3.0)	*0.141* (3.918, 2)
Clopidogrel	0 (0.0)	5 (18.5)	3 (9.1)	*0.314* (2.315, 2)

*Abbreviations*: *ACEIs* Angiotensin-converting enzyme inhibitors, *ARBs* Angiotensin-receptor blockers, *CCBs* Calcium channel blockers, *NSAIDs* Non-steroidal anti-inflammatory drugs, *LMWH* Low molecular weight heparin, *PPIs* Proton pump inhibitors

The sensitivity of the cardiologist in the ECG and presenting symptoms diagnosis of hyperkalemia was 35.8% as hyperkalemia was successfully detected in 24 out of 67 hyperkalemic patients. In contrast, the sensitivity was 28.4% (19 out of 67) for the emergency physician. There was 86.6% agreement between the readers on the presence of hyperkalemia based on patients’ ECGs and presenting symptoms, with κ of 0.694, which suggests substantial inter-rater reliability for diagnosing hyperkalemia. In cases of severe hyperkalemia with potassium levels of more than 6.5 mmol/L, the readers’ sensitivities increased to be 51.5% (17 of 33) and 42.4% (14 of 33) for the cardiologist and emergency physician, respectively.

The mean (±SD) time to initial treatment of hyperkalemia was 63.8 ± 31.5 min and ranged from 15 to 110 min. Table 5 shows the correlations of the time to hyperkalemia treatment and serum potassium levels with ECGs’ changes suggestive of hyperkalemia and patients’ vital signs and laboratory data at the time of presentation. The time to hyperkalemia treatment was not significantly correlated with serum potassium levels, ECG alterations, patients’ vital signs, and laboratory values (*p>0.05*).

**Table 5:** Correlations of time to treatment and serum potassium levels with ECGs’ alterations, patients’ vital signs, and their laboratory values

	Time to hyperkalemia treatment^**a**^		Serum potassium levels	
	Pearson r	***p-value***	Pearson r	***p-value***
**ECG changes suggestive of hyperkalemia**				
PR interval (msec)	−0.209	*0.130*	0.283*	***0.038***
QRS duration (msec)	−0.033	*0.793*	0.361**	***0.003***
QTc interval (msec)	0.187	*0.134*	0.123	*0.324*
RR interval (msec)	−0.163	*0.188*	0.003	*0.984*
Presence of peaked T wave^b^	−0.035	*0.779*	0.242*	***0.041***
**Vital signs at the time of emergency presentation**				
Temperature (°C)	0.057	*0.647*	−0.048	*0.702*
Heart rate (beats per min)	0.155	*0.210*	0.002	*0.990*
Respiratory rate (breaths per min)	0.081	*0.512*	0.071	*0.568*
Systolic pressure	−0.133	*0.285*	−0.224	*0.068*
Diastolic pressure	0.073	*0.557*	−0.234	*0.057*
Pulse oximeter (O_2_ saturation)	−0.138	*0.267*	0.000	*0.997*
**Serum laboratory values at the time of emergency presentation**				
Potassium levels	−0.044	*0.722*	1	*-*
Creatinine levels (μmol/L)	0.183	*0.138*	0.347**	***0.004***
BUN (mmol/L)	0.166	*0.180*	0.312**	***0.008***
Ph	0.100	*0.467*	−0.259	*0.056*
PCO_2_ (mmHg)	−0.067	*0.625*	−0.101	*0.462*
HCO_3_ (meq/L)	0.018	*0.895*	−0.240	*0.078*
BE (base excess) (meq/L)	−0.056	*0.692*	−0.158	*0.259*
Troponin (ng/ml)	0.160	*0.415*	−0.281	*0.147*
CK-MB (IU/L)	−0.040	*0.832*	0.148	*0.428*
CK (U/L)	0.131	*0.481*	0.373*	***0.039***

^a^Time to hyperkalemia treatment was calculated by the difference between the triage time and the first hyperkalemia therapy administration

^b^Peaked T wave was coded with one when it is present and zero when it is absent

Bold indicates significant statistical correlations: *Correlation is significant at the 0.05 level, while **correlation is significant at the 0.01 level

Among ECGs criteria, PR interval, QRS duration, and peaked T wave abnormalities were significantly correlated with serum potassium levels in positively linear manners (*r* = 0.283, *p=0.038*; *r* = 0.361, *p=0.003*; *r = 0.242*, *p=0.041*, respectively). However, the correlations were weak. These correlations indicate that increased serum potassium level was associated with a slight increase in PR interval and QRS duration and with the presence of peaked T waves. Whereas serum potassium levels did not significantly correlate with QTc intervals, RR intervals, and the vital signs of hyperkalemic patients (*p>0.05*). Serum potassium levels were positively correlated with serum levels of creatinine (*r* = 0.347, *p=0.004*), BUN (*r* = 0.312, *p=0.008*), and CK (*r* = 0.373, *p=0.039*). Similarly, QTc intervals on ECGs were positively correlated with serum BUN levels (*r* = 0.418, *p<0.001*) and negatively with Ph (*r* = 2.80, *p=0.040*).

## Discussion

Hyperkalemia is a common electrolyte imbalance in adults with a potentially peri-arrest risk, but of reversible possibility when diagnosed and managed in time. This study highlights the challenges associated with the diagnosis and management of hyperkalemia. Hyperkalemic patients in our study tend to be older adults and elderly and suffer from several comorbidities, such as HTN, DM, and chronic kidney disease. Besides, drugs inducing hyperkalemia were common, including analgesics, beta-blockers, CCBs, ACEIs, and ARBs. Given the non-specific clinical presentations of hyperkalemia, and about one-quarter of our patients were asymptomatic, we evaluated ECG as an attainable test to raise the possibility of hyperkalemia. The results indicated a poor sensitivity of initial ECG and presenting symptoms in detecting hyperkalemia as it ranged between 0.28 and 0.36 and improved minimally when potassium ≥6.5 mmol/L with peaked T wave was significantly more observed than in mild and moderate hyperkalemia. Thus, the absence of ECG alterations suggestive of hyperkalemia should not lower the physician’s suspicion of the presence of hyperkalemia in high-risk patients. Also, a mean delay of 1 h from triage to initial hyperkalemia treatment was observed, which is an alarming finding.

Hyperkalemia is usually multifactorial in etiology, and we did not attempt to investigate the specific causes for each case of hyperkalemia. However, in this study, our patients with elevated potassium levels had one or more conditions causing hyperkalemia such as comorbid HTN, DM, or CKD, and high rates of certain medications use, including non-steroidal anti-inflammatory drugs (NSAIDs) beta-blockers, CCBs, ACEIs, and ARBs. These findings are concordant with previous reports on the risk factors of hyperkalemia [6, 14, 15, 47–53]. Our study as well reported high rates of statins and PPIs to use within hyperkalemic patients. Previous reports indicated that statin use as a cytochrome P450s 3A4 inhibitor, especially in combination with antihypertensive medications, could contribute to acute kidney injury and hyperkalemia [54, 55]. Previous investigators also observed elevated serum potassium levels among PPI users [56, 57]. However, as this is a cross-sectional study, we could not establish causality associations between statins and PPIs with hyperkalemia. Our population’s high rates of insulin use represent a marker for more advanced contributing comorbidities such as DM, which may induce hyperkalemia through developing type IV renal tubular acidosis. Thus, we could conclude that the patients with multiple comorbidities such as HTN, DM, or CKD and those on hyperkalemia-inducing medications were eligible for regular monitoring of electrolytes disturbances.

ECGs had abnormalities consistent with hyperkalemia among around two third of the studied patients with hyperkalemia, and the most common alterations were elevations of T wave amplitude and QRS duration. As well, increased PR interval and QRS duration and presence of peaked T wave were correlated with serum potassium levels. These findings align with previous reports of a higher frequency of ECG alterations suggestive of hyperkalemia with elevated serum potassium levels [33, 58]. Trail has found that ECG disturbances, including peaked T waves and an increase in the duration of the QRS complex, were associated with hyperkalemia and more evident with a serum potassium level of ≥7.8 mEq/L [34]. T wave in ECG occurs due to repolarization of ventricles, whereas QRS duration represents the time for ventricular depolarization, and PR interval represents the time between atrial depolarization and ventricular depolarization. Hyperkalemia causes an increase in the velocity of phase 3 of the action potential, which is associated with the peaking of the T wave. Also, hyperkalemia causes a decrease in the resting membrane potential of myocardial cells with less negativity which causes conduction defects and prolongation of the PR intervals and QRS complexes [59–61].

Varga et al. reported the QRS widening, peaked T waves, first-degree heart block, and bradycardia as the most frequent ECG alterations suggestive of hyperkalemia in severely hyperkalemic patients with serum potassium levels of >7 mmol/L (31.6%, 18.4%, 18.4%, 18.4%, respectively) [58]. These ECG alterations were significantly more common among severely hyperkalemic patients than in normokalemia patients (8.2, 4.7, 7.1, and 6.5%, respectively) [58]. Hicks, in his case report, assessed hyperkalemia-associated ECG findings in a 34-year-old female with DM, abnormal cardiac rhythm, and no known history of renal failure presented to the ED [62]. The most significant findings were peaked T waves and widening QRS complex with a potassium level of 7.6 mmol/L [62]. Peaked T waves could be considered one of the typical and earliest ECG signs of elevated serum potassium levels [2, 22, 32–34, 62, 63].

Other ECG alterations suggestive of hyperkalemia in our study included flattening and disappearance of P waves, RBBB, and ST elevations were observed. Similarly, in a clinical review, a woman presented to the ED with respiratory distress and altered mental status and had an elevated serum potassium level of 9.6 mmol/L; her ECG recorded ST elevations, RBBB, and loss of P wave amplitude [64]. ST segment is the state of the ventricles between repolarization and depolarization. ST elevation had been previously linked to hyperkalemia and called “pseudo-infarction”; therefore, hyperkalemia is a potential differential diagnosis for the cause of elevations in the ST segments [65]. However, the mechanism of ST segment elevation due to hyperkalemia is not already known [64]. Also, ST depression and shortening of the QTc interval had been reported in several investigations as manifestations of hyperkalemia [23, 66, 67].

Most studies suggested an association between lower potassium levels and a higher risk of atrial fibrillation [68–71]. However, our results reported atrial fibrillation as the most common arrhythmia observed among hyperkalemic patients. This finding is concordant with Varga et al.’s findings that aThe trail fibrillation was more prevalent in severely hyperkalemic patients than normokalemia patients [58]. We attribute these results to the synergistic effect of CHF and CKD, which often present in patients with high serum potassium levels. Hyperkalemia and CHF are common in chronic kidney disease, and CHF could cause atrial fibrillation. Thus, atrial fibrillation occurs not as the result of hyperkalemia but rather as the consequence of illnesses often associated with hyperkalemia.

ECG is an inexpensive, broadly available, and easily attainable test. There have been conflicting reports about its sensitivity and specificity to capture elevated serum potassium levels [22, 29–32, 37, 63]. Our study indicated poor sensitivity of initial ECG and clinical presentation in detecting hyperkalemia as ranged between 0.28 and 0.36. These results are concordant with previous studies showing that physicians’ ability to predict hyperkalemia from the ECG was low with sensitivities between 0.43 and 0.34, and experienced readers’ ability to predict the severity of hyperkalemia was likewise poor [22, 32]. Similarly, Rafique et al. reported a mean sensitivity of 0.19 (± 0.16) for the emergency physicians detecting hyperkalemia based on the ECG, and this sensitivity improved to 0.29 (± 0.20) in severe hyperkalemia [39]. Varga et al. captured ECG alterations suggestive of hyperkalemia among 46% of the hyperkalemic patients, and surprisingly 24% of normokalemia patients exhibited such ECG alterations [58]. Thus, based on ECG analysis and with or without presenting symptoms knowledge, the physician could not confirm or exclude hyperkalemia, and serum laboratory tests should be conducted for accurate hyperkalemia diagnosis. Montague et al. had conducted a study on ninety patients diagnosed with hyperkalemia as serum potassium of ≥6 mmol/L [33]. The authors reported that the probability of ECG changes increased with increasing potassium levels, but the sensitivity and specificity of ECG changes in diagnosing hyperkalemia were poor [33]. It could be concluded that the management of hyperkalemia should be guided by the clinical scenario and serial laboratory potassium measurements, and the absence of ECG alterations suggestive of hyperkalemia should not lower the physician’s concern for the presence of hyperkalemia in high-risk patients.

Although the lack of sensitivity in detecting hyperkalemia based on ECGs ultimately depends on physicians’ interpretations, other confounding factors could not be excluded. First, the possible effects of other electrolytes, such as calcium and magnesium, in mitigating the ECG changes suggestive of hyperkalemia as proposed by prior investigators [31, 72–74]. Second, 64% of our patients suffered from CKD, and about one third of participants were on regular dialysis, which could cause the non-specificity of ECG abnormalities. It was reported that hemodialysis patients with hyperkalemia were less likely to show ECG changes despite the risk of suddenly developing arrhythmias as the myocytes were less sensitive to electrolyte changes in these patients; therefore, hyperkalemia did not manifest in them its typical forms [25, 37, 75]. Third, the rate of increase in serum potassium levels could affect the development of ECG changes [1, 21, 31]. As the velocity of serum potassium concentrations risen was unknown to the readers, their insensitivity could be attributed to the slowly rising potassium levels, especially in the setting of CKD. Fourth, patients’ medications such as digitalis could have interacted with the effects of electrolytes on myocytes and masked the effects of hyperkalemia. Lastly, metabolic acidosis and ischemia could be associated with arrhythmias and ST and T wave alterations in the patterns suggestive of hyperkalemia [76]. Although including serum markers of cardiac ischemic injury and ABGs for acidosis detection, the absence of abnormalities in these serum markers does not exclude them as confounding factors. There is also the potential that elevated serum potassium levels may potentiate arrhythmias that could be attributable to other causes. However, these possibilities could not be ruled out as they are part of clinical practice.

One of our most striking findings was that the meantime from triage to initial hyperkalemia treatment of more than one hour. Freeman et al. investigated the possible effects of presentations and ECGs on triage time to the initial hyperkalemia management [22]. The authors found that most hyperkalemic patients waited for a median of 2 h from triage to initial treatment, even though ECG was performed before the laboratory serum potassium measurement [22]. Also, the delay in hyperkalemia treatment was reported among hospitalized patients, with approximately 2 h delays from laboratory notification of potassium to initiation of treatment [2].

In our study, we observed some behaviors in hyperkalemia treatment, which would explain the delays. In some cases, the initial physician response to an unexpectedly elevated potassium level was to repeat the serum potassium test and obtain intravenous access. In other cases, difficulties in performing intravenous access were documented, which is expected for a population including 64% suffered from CKD and 24% of patients on regular dialysis. However, other treatments should be considered in patients with difficult intravenous access, including high-dose inhaled beta-agonist therapy or direct intravenous injection of hyperkalemia therapies in a life-threatening situation.

### Strengths, implications, and limitations of this study

The strengths of this study included the prospective nature of data collection and processing, uniform data collection, consistent definitions applied, and detailed periodic review of the abstracted data, which support the integrity and validity of the collected data. Also, the ECGs were conducted simultaneously within 1 h of serum potassium level measurement and before initiation of therapy, which assured tight data pairing. In addition, the initial ECGs and patients’ symptoms at the time of ED presentation were interpreted by an emergency physician and a cardiologist to predict hyperkalemia while blinding to all laboratory values, study design, patients’ diagnoses and comorbidities, and each other’s readings to reduce bias. Since the readers considered the possibility of non-hyperkalemia diagnoses when interpreting the initial ECGs, our study was more realistic and similar to the clinical emergency practice and reduced the readers’ reported sensitivities. Also, this study highlighted the delay in hyperkalemia treatment registration that might be considered a failure due to a missed diagnosis based on clinical presentations and initial ECG alterations.

All these factors contributed to a more robust study, which supports the conclusions of previous reports that the clinical presentations and ECGs are not reliable tools in the diagnosis of hyperkalemia [22, 32, 39, 58]. Although our study indicated that the recognition of hyperkalemia is challenging and the initiation of appropriate therapy is frequently delayed, it had been suggested by Riccardi et al. that initiation of intravenous calcium gluconate as a life-saving treatment to stabilize the cardiac membrane in suspected hyperkalemia before laboratory confirmation would be advisable [77]. However, previous investigations reported that the empiric hyperkalemia therapy based on ECG solely was associated with the mistreatment of approximately 15% of patients [32]. Thus, given the non-specific nature of the patients’ clinical presentations and the variability of ECG presentations of hyperkalemia, it is prudent to delay hyperkalemia treatment in relatively stable patients until laboratory confirmation of hyperkalemia. In unstable patients, intravenous calcium gluconate as an empirical treatment for hyperkalemia could be initiated based on ECG alterations solely. Also, the absence of ECG alterations suggestive of hyperkalemia should not affect the physician’s suspicions of hyperkalemia in high-risk patients. In light of these facts and the reported delays for the initiation of hyperkalemia therapy, technological advancements should be considered in high-risk patients, such as using finger-stick testing [78] and incorporating artificial intelligence into the ECGs [78].

Our study has several limitations. Firstly, the inherent limitations associated with the cross-sectional design could not establish causality inference. This study did not include a control group with normal serum potassium levels to compare, limiting our results’ internal validity; therefore, we were unable to calculate the specificity and predictive values of ECG in diagnosing hyperkalemia. Also, it was conducted on a relatively small sample size of patients admitted to emergency care and a small number of ECG readers. Moreover, a sample size calculation was not done. This study was conducted at a single center and on a narrow ethnicity of patients, which limits the generalizability of our results and conclusions beyond our patients. Hence, a larger number of hyperkalemic patients with different ethnicities and appropriate disease prevalence rates calculation with involving more evaluators would help improve the robustness of the study. Also, there might have been human error in conducting and interpreting ECGs, which was not being considered. However, we uploaded a color copy of the ECG with the highest quality to reduce misinterpretations, and this study included a relatively small number of ECGs to reduce the possibility of readers’ fatigue.

Another limitation is the interpretation of the initial ECGs solely as isolated tracings in this study without comparing them with the previous ECG tracings. Also, the notations of dynamic ECG changes during the emergency course were not recorded and were unavailable to the readers to interpret the initial ECGs. However, in the actual practice, a comparison of the initial ECGs with prior ECGs might contribute to the delay in hyperkalemia therapy administration. Also, there is potential for confounding factors in the interpretation of ECGs that might modify the ability of physicians to predict hyperkalemia since several ECG alterations could be attributed to other causes than hyperkalemia or ECG changes due to other conditions might mask the ECG signs of hyperkalemia. These confounding factors included other electrolyte abnormalities, such as calcium and magnesium, CKD, hemodialysis, and drugs such as digitalis, metabolic acidosis, and myocardial ischemia that might mask the ECG changes suggestive of hyperkalemia. However, this study collected and included the patients’ comorbidities, used medications, ABG measurements, and cardiac ischemic injury serum markers. Finally, the time to hyperkalemia treatment was calculated based on the triage time and the first hyperkalemia therapy administration by the nurse. This methodology could mask other potential causes for delay in hyperkalemia treatment, such as the time spent before triage and the time between physician order and nursing administration of treatment. Further prospective larger-scale studies examining the ECG alterations suggestive of hyperkalemia in hyperkalemic patients compared with normokalemia individuals with attention to other electrolytes levels would be needed to confirm our findings.

## Conclusions

This study results with previous studies findings have suggested the wide variability of clinical presentations and ECG abnormalities among patients with hyperkalemia, and the physicians had poor sensitivity in detecting hyperkalemia using the ECGs and clinical presentations. The most frequent signs observed on ECGs and well known to physicians were peaked T waves, QRS widening, prolongation of the PR interval, and absence of P wave. Also, hyperkalemia might mimic acute myocardial infarction with ST elevations. Peaked T wave abnormality was observed in patients with severe hyperkalemia. However, there is no clear evidence supporting ECG use to guide hyperkalemia management, and laboratory tests should be conducted to detect the mild to moderate rise in serum potassium levels which might get missed using ECG. Thus, the ECG has little value in the diagnostic algorithm of hyperkalemia and should be used with caution. The absence of ECG alterations suggestive of hyperkalemia could not be relied on to rule out hyperkalemia and should not lower the suspicion of hyperkalemia, especially in high-risk patients. An accurate laboratory-confirmed diagnosis of hyperkalemia was warranted before initiation of treatment in stable patients. Nevertheless, ECG changes suggestive of hyperkalemia should draw attention among unstable patients in emergencies.

Since the non-specific clinical presentations of hyperkalemia and uncertainty of ECG diagnosis of hyperkalemia, our data suggests the importance of regular electrolytes monitoring among older adults and elderly patients on hyperkalemia-inducing medications and those with renal and cardiac diseases, preferably within the primary care settings in non-urgent situations. Given the inherent delays in initiation of hyperkalemia treatment which is an alarming finding, other methods for early detection and intervention in hyperkalemia should be considered in the ED, including rapid finger-stick testing and using artificial intelligence with the ECGs. Also, intravenous calcium gluconate administration could be considered in unstable patients with ECG alterations suggestive of hyperkalemia for cardiac resuscitation and prevent arrhythmias as its benefits outweigh the risks. Further prospective more extensive studies are needed to assess the stability and progression of ECG changes in patients with hyperkalemia.

## Data Availability

The datasets used and analyzed during the current study are available from the corresponding authors on reasonable request.

## Abbreviations

ED: Emergency department
ACEIs: Angiotensin-converting enzyme inhibitors
ARBs: Angiotensin-receptor blockers
CHF: Congestive heart failure
ECG(s): Electrocardiogram(s)
QTc: QT corrected
KAUH: King Abdullah University Hospital
JUST: Jordan University of Science and Technology
BUN: Blood urea nitrogen
CK-MB: Creatine kinase-MB
CK: Creatine kinase
RBBB: Right bundle branch block
LBBB: Left bundle branch block
IRB: Institutional Review Board
ACC: American College of Cardiology
SPSS: Statistical Package for the Social Sciences
κ: kappa
HTN: Hypertension
DM: Diabetes mellitus
CKD: Chronic kidney disease
IHD: Ischemic heart disease
COPD: Chronic obstructive pulmonary disease,
CCBs: Calcium channel blockers
NSAIDs: Non-steroidal anti-inflammatory drugs
LMWH: Low molecular weight heparin
PPIs: Proton pump inhibitors
SVT: Supraventricular tachycardia
